# Testing the Performance of the Azo-Polyimide Supramolecular Systems as Substrate for Sensors Based on Platinum Electrodes

**DOI:** 10.3390/ma16144980

**Published:** 2023-07-13

**Authors:** Ion Sava, Mihai Asandulesa, Andreea Irina Barzic, Raluca Marinica Albu, Iuliana Stoica

**Affiliations:** “Petru Poni” Institute of Macromolecular Chemistry, Aleea Gr. Ghica Voda 41A, 700487 Iasi, Romania; asandulesa.mihai@icmpp.ro (M.A.); irina_cosutchi@yahoo.com (A.I.B.); albu.raluca@icmpp.ro (R.M.A.)

**Keywords:** supramolecular structure, polyimide film, azo monomer, mechanical behavior, AFM, dielectric properties

## Abstract

Azo-polyimide films with supramolecular structure were obtained by casting onto glass plates a mixture based on polyamidic acid and different quantities of azochromophore, followed by thermal treatment to realize the final azo-polyimide structure. The dielectric characteristics of the supramolecular structure of polymer films were investigated by broad-band dielectric spectroscopy measurements at different temperatures and frequencies. The free-standing films proved to be flexible and tough and maintained their integrity after repeated bending. The work of adhesion at the polymer/platinum interface was calculated after the evaluation of the surface energy parameters before and after plasma treatment. Atomic force microscopy was used to image the surface morphology, the evolution of the roughness parameters, and the adhesion force between the platinum-covered tip and the polymer surface, registered at the nanoscale with the quantity of the azo dye introduced in the system. The simulation of the columnar growth of a platinum layer was made to provide information about the deposition parameters that should be used for optimal results in the deposition of platinum electrodes for sensors.

## 1. Introduction

Azopolymers are compounds that consist of azobenzene molecules linked or scattered in a polymer. Their properties are due to the behavior of the azobenzene molecules when exposed to UV and visible light. It is well known that the azobenzene units can be in a thermodynamically stable *trans* and a less stable *cis* isomer. In the process of irradiation with linearly polarized light of adequate wavelength, the azobenzene groups undergo many reversible *trans*-to-*cis* photoisomerization processes and produce photoinduced optical anisotropy as a result of the interaction of azobenzene molecules with the actinic electric field vector [1,2,3,4]. Various methods are used for the introduction of azobenzene into polymers. The host–guest system, which can be obtained by mixing a guest chromophore (azobenzene compound) in a host polymer matrix, is the most simple. This process is suitable from the point of view of preparation but can present inconveniences regarding the low solubility of the chromophore in the host polymer, the aggregation of the dye molecules, the separation of two components, and even the evaporation of active molecules from the films [5,6,7,8,9]. The other way to obtain azopolymers is by adding chromophores covalently to the chain of the polymer. Thus, it will be realized that photoinduced anisotropy is stable and produces efficient surface relief grating (SRG) formation [10,11,12,13,14]. This method encountered difficulties regarding synthetic procedures to obtain the polymers, which are eliminated by using the host–guest system. At the same time, the noncovalent intermolecular interactions are certified to be suitable for operative SRGs [15,16,17,18,19]. Azo-polyimides, which are represented by polyimides containing azobenzene units, are of special interest due to their use as materials for a lot of applications, such as dielectric films for the electronic industry, orientation layers in liquid crystal materials, heat-resistant composites, adhesives for aerospace domains, and gas separation membranes [20,21,22,23,24]. The polyimides were selected due to their high glass transition temperature, which is indispensable for the improved temporal and thermal stability of the photoinduced chromophore alignment in the dark. Consequently, they are assessed as being very good hosts for supramolecular azo systems devoted to the inscription of stable optical anisotropy. Ten years ago, the scientific group of E. Schab-Balcerzak reported for the first time the obtaining of hydrogen-bonded supramolecular azo-polyimides [25,26,27,28,29].

Recently, we reported supramolecular azo-polyimides with high thermal stability and good behavior for the formation of high SRG by using an innovative method [30,31]. This method consists of adding the azo compound to the polyamidic acid, followed by thermal treatment to realize the final imide structure. Such supramolecular architectures could be the key element in the production of modern sensor devices. They have the advantage of being capable of adapting their bulk and surface characteristics through the incorporation of azo moieties. In a previous study, we showed that phase mask laser exposure of other polyimide structures leads to important surface morphology and wettability changes as a function of the material composition [32]. To our knowledge, there are no other reports on azo-polyimides characterization for use as substrates for sensors. During the device fabrication, the polymer layer must withstand a certain level of deformation, so the mechanical resistance should be analyzed. Moreover, the dielectric response under the electric field should be suitable for minimizing the resistance-capacitance delay and allowing a faster propagation of the signal in the metallic conductor lines. This is why it is recommended that the polymer substrate have a low dielectric constant (below 3) [33]. Another essential point that dictates the reliability of the device is the compatibility of the polymer and metal parts. To ensure this, it is mandatory to evaluate the surface properties of the flexible substrate in terms of morphology, wettability, and how they impact the polymer adhesion with the metal electrode. The study of these correlated bulk and surface properties of azo-polyimides for sensor applications has not yet been developed in the literature.

The current article continues the previous idea of testing azo-polyimides for sensors made of another type of metal, namely platinum. The research strategy was focused not only on the azo-polyimide surface properties but also on bulk mechanical and dielectric testing. The surface analysis involves wettability experiments to assess the interfacial work of adhesion. The surface morphology was visualized via atomic force microscopy (AFM). The local mechanical properties were evaluated from the scanning probe spectroscopy measurements performed at the nanoscale at the interface of the platinum and the polymer surfaces. The simulation of the columnar growth of a platinum layer was made to provide information about the deposition parameters that should be used for optimal results. This has not been reported in the literature. All obtained data were correlated with the amount of azo monomer incorporated, which helped to design new materials with an appropriate balance of properties as demanded for sensors.

## 2. Materials and Methods

**Scheme 1:**
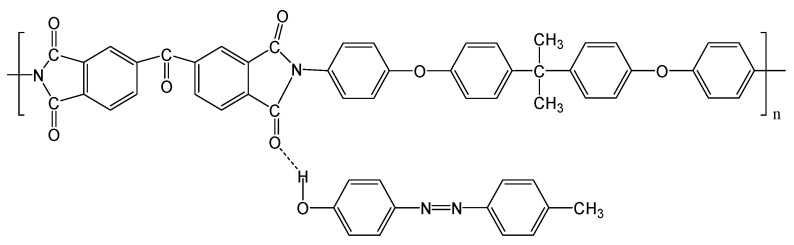
The structural formula of the guest–host polyimide films.

**Figure 1:**
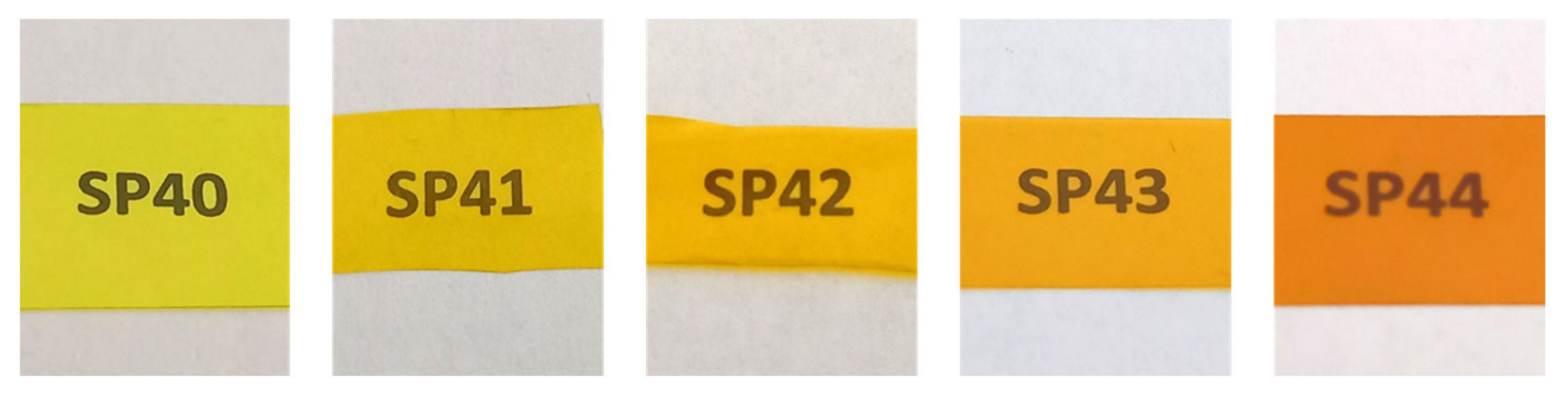
The photographs of the **SP40**–**SP44** azo-polyimide films placed on a sheet of white paper on which the name of each sample was printed in black.

Supramolecular structures (**SP**) were obtained by mixing 4-[(4-methylphenyl)diazenyl]phenol (Az(CH_3_)) with an aromatic polyamidic acid solution [30]. Polyamidic acid (PAA) was synthesized by the polycondensation reaction between 4,4′-(4,4′-isopropylidenediphenyl-1,1′-diyldioxy)dianiline (6HDA) and benzophenonetetracarboxylic dianhydride (BTDA) in dimethylacetamide at a concentration of 15%. The molar ratio between polyamidic acid and azo monomer was 1:0.125 (**SP41**), 1:0.25 (**SP42**), 1:0.5 (**SP43**), and 1:1 (**SP44**). The abridgement **SP40** was utilized for primary polyimide film based on BTDA and 6HDA. By casting guest–host PAA solution on a glass plate, flexible films were obtained, leading to the final imide structure after thermal treatment (Scheme 1). In Figure 1, the photographs of the thus obtained **SP40**–**SP44** azo-polyimide films are presented and placed on a sheet of white paper on which the name of each sample is printed in black. It can be easily observed that the pristine polyimide film **SP40**, based only on BTDA and 6HDA, has a bright yellow hue. As the molar ratio between polyamidic acid and azo monomer increases from 1:0.125 (**SP41**) to 1:1 (**SP44**), the color of the films evolves from a dark yellow tone to an orange shade. In addition, it can be noticed that all the films retain their transparency even after adding a considerable amount of azochromophore.

Dielectric properties were investigated with a Novocontrol Concept 40 Broadband Dielectric Spectrometer (Novocontrol Technologies GmbH & Co. KG, Montabaur, Germany). The electrical field oscillations were generated by an Alpha-A high-performance frequency analyzer. The dielectric spectra were recorded at room temperature in a frequency window between 1 Hz and 1 MHz. The measurements were provided at temperatures between −120 and 250 °C. Circular samples of 2 cm in diameter cut out of previously obtained free-standing films were placed between two gold-plated flat electrodes and then placed in the active BDS sample cell system. To prevent moisture absorption, measurements were conducted in a dry nitrogen environment. Stress-strain measurements were performed on dumbbell-shaped samples cut at a dimension of 20 mm × 5 mm from the thin films, on a TIRA test 2161 apparatus (Maschinenbau GmbH, Ravenstein, Germany). All the measurements were accomplished at an extension rate of 50 mm/min at room temperature. The final value obtained was the average of three measurements. The wetting features of the compound surface films were evidenced by means of water and formamide contact angle experiments. The measurements were performed on rectangular specimens of 1 cm × 3 cm using a device designed in our laboratories and were repeated five times by employing liquid drops of 5 µL volume. The measuring error of the contact angle on the samples was ±1°, leading to a relatively low statistical error (standard deviation). Plasma treatment of the polymer layers was undertaken on an RPS40 system (ROPLASS) for 2.5 min. Atomic force microscopy (AFM) measurements were performed on an NTEGRA multifunctional scanning probe microscope (NT-MDT Spectrum Instruments, Zelenograd, Moscow, Russia), using Nova 1.1.1.19891 software for data acquisition and analysis. The morphology was registered on a square area of 1 cm × 1 cm in tapping mode using an NSG10 cantilever (with the following characteristics: length—95 ± 5 µm, width—30 ± 3 µm, thickness—2.0 ± 0.5 µm, tip curvature radius—6–10 nm, resonant frequency—236 kHz) from NT-MDT Spectrum Instruments, Zelenograd, Moscow, Russia. The DFL–height curves (the plots of the cantilever’s deflection (DFL signal) vs. the tip–sample distance during the approach-retraction movement) were acquired through the AFM force curve spectroscopy (FCS), in contact mode, using a CSG30/Pt cantilever (with the characteristics: length—190 ± 5 µm, width—30 ± 3 µm, thickness—0.5 ± 1.5 µm, typical tip curvature radius—35 nm, resonant frequency—54 kHz, force constant—1.6 N/m) from NT-MDT Spectrum Instruments, Zelenograd, Moscow, Russia. The value of the adhesion force was calculated from the retraction DFL–height curve, according to Hooke’s law: Fadh = −k·Δx, taking into account that k is the force constant of the cantilever and Δx is the deviation of the cantilever at the final contact state before the jump of the tip from contact. The Gwyddion 2.56 software was used to simulate the columnar surface deposition of the platinum nanoparticles.

## 3. Results and Discussion

The utilization of the analyzed systems for implementation in electronic products, such as flexible supports for sensors, imposes deep knowledge of the key bulk and surface properties. The response of the polyimide-based films under electrical field influence must be examined to probe the insulation characteristics and polarization mechanisms that are affecting the level of the signal passing through the deposited metal electrode. Moreover, the mechanical resistance of the polymer substrate must be demonstrated to check this applicative criterion. Another essential aspect resides in the surface characteristics (morphology and wettability), which in turn influence the polymer layer’s interaction with the metallic electrode. All mentioned properties are discussed in the next sections of the paper.

### 3.1. Dielectric Properties

The effect of AzoCH_3_ on dielectric characteristics such as dielectric constant, dielectric loss, and relaxation properties of **SP** polymers was studied at frequencies ranging from 1 Hz to 1 MHz and temperatures ranging from −150 °C to +270 °C. The dielectric relaxation measurement at different frequencies of **SP** with varied AzoCH3 concentrations provides information regarding polar group dynamics and molecular mobility.

**Figure 2:**
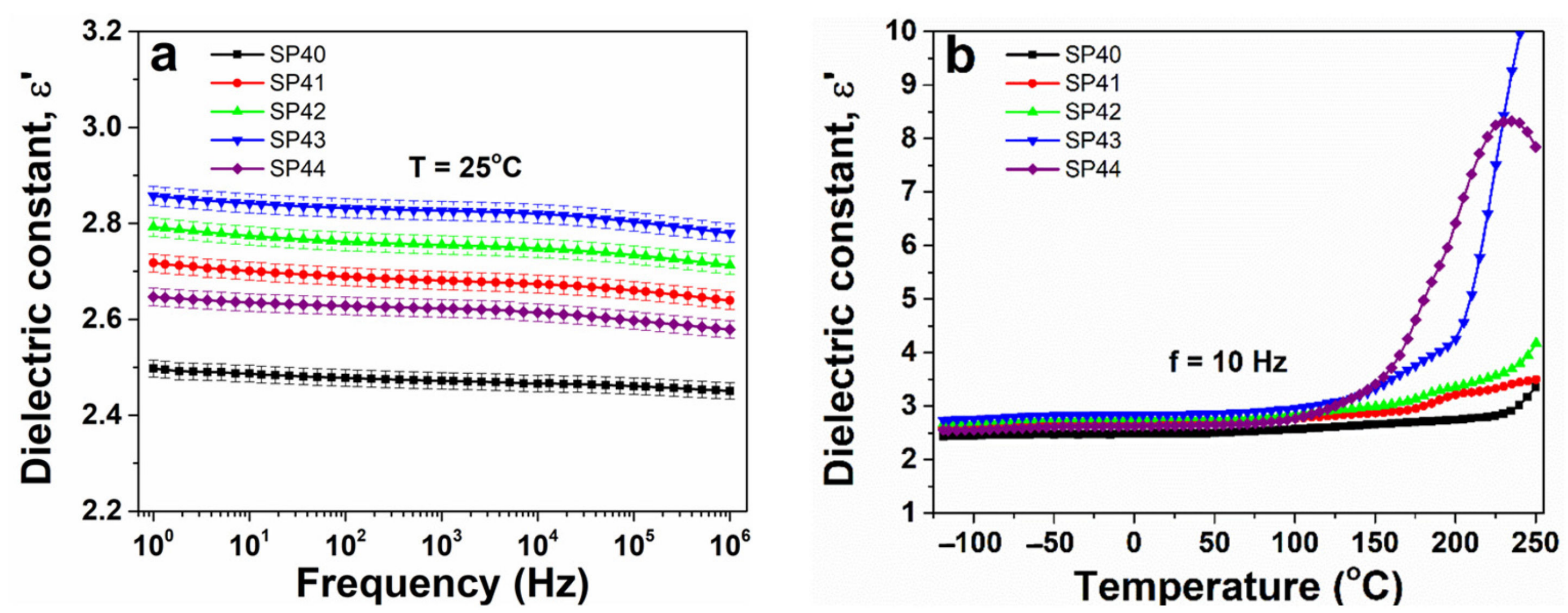
The behavior of dielectric constant with frequency at room temperature (**a**) and with temperature at 10 Hz (**b**) for the pristine polyimide and supramolecular films.

The behavior of the dielectric constant, *ε*′, as a function of frequency and temperature, is shown in Figure 2a and Figure 2b, respectively. As can be seen in Figure 2a, the dielectric constant had a modest decrease with increasing frequency, as generally observed for polyimide-based materials [34,35]. The incorporation of the azo monomer increased the value of the dielectric constant, depending on the ratio between the two components. By using a molar ratio of 1:0.125, 1:0.25, and 1:0.5, the dielectric constant slightly increased at 2.7, 2.8, and 2.85, respectively, compared with the polymer reference **SP40**, which has the smallest value (e.g., *f* = 1 Hz, ε′ = 2.5). Following the increase in the molar ratio to 1:1, however, *ε*′ of the **SP44** polymer is moderately enhanced up to 2.65. At this point, we may notice that the optimal molar ratio within the highest enhancement of the dielectric constant is 1:0.5. We believe that the increase in dipolar activity in the bulk of final products is mostly due to the existence of polar molecules induced by hydrogen bonds. The latter were assessed when the azo monomer and polyimide components were mixed in various ratios. A slightly increased dielectric constant can be evidence of the existence of polar molecules due to hydrogen bonds compared to pristine polyimide film.

The behavior of the dielectric constant with temperature at a frequency of 10 Hz is shown in Figure 1b for all investigated samples. The dielectric constant of supramolecular structures is relatively low and almost unchanged at temperatures between −120 and 100 °C. With further increases in temperature, the cooperative motions of chain segments from the polymer backbone were thermally activated, and consequently, the magnitude of *ε*′ increased rapidly, being correlated with the molar ratio of the guest–host components. Therefore, if the increase in the dielectric constant is moderate at lower ratios (1:0.125 and 1:0.25) and at higher temperatures is close to the pristine polyimide film (above 225 °C), by increasing the AzoCH_3_ content to 1:0.5 and 1:1 molar ratios, the increase in the dielectric constant is sharp and starts at temperatures of 150 °C and 130 °C, respectively. This is very important for proving that the azo monomer influences in great measure the behavior of the dielectric constant by increasing the temperature.

**Figure 3:**
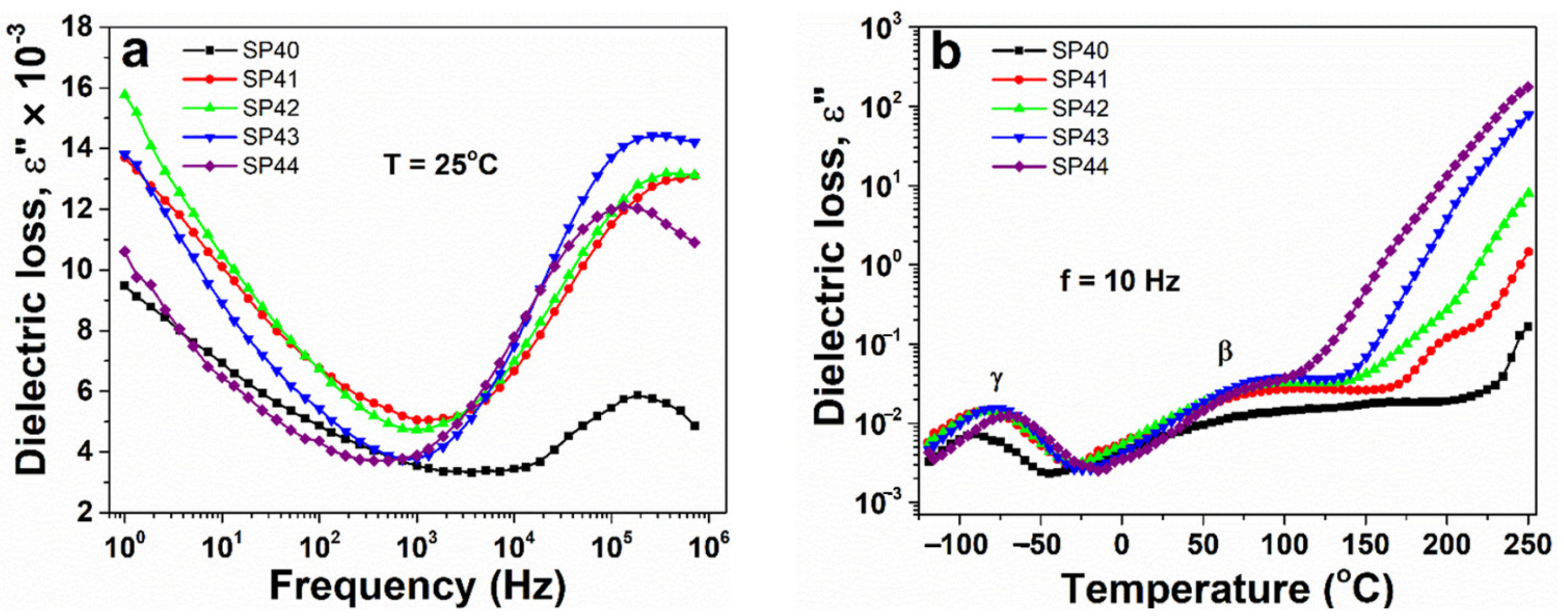
The behavior of dielectric loss with the applied field frequency at room temperature (**a**) and temperature at 10 Hz (**b**) for the studied materials.

The dielectric loss of polymers, *ε*″, was low, below 0.015 (Table 1), and slowly decreased with increasing frequency, irrespective of the ratio used, attaining lower values around 10^3^ Hz and then increasing again (Figure 3a).

The temperature dependences of dielectric loss selected at 10 Hz reveal the presence of secondary-type relaxation dipolar processes: (i) The γ-relaxation is observed at temperatures between −120 °C and −20 °C, and (ii) the β-relaxation at temperatures between 40 and 100 °C. In addition, at temperatures higher than 150 °C, the dielectric loss of polyimide films based on supramolecular structures is higher, mostly probably due to the presence of charge carriers (Figure 3b). Coming back to the findings from Figure 3a, we may assume that the low-frequency dispersion occurring below 10^3^ Hz is the head part of the β-relaxation signal, while the dielectric signal between 10^3^ Hz and 10^6^ Hz is attributed to γ-relaxation.

**Figure 4:**
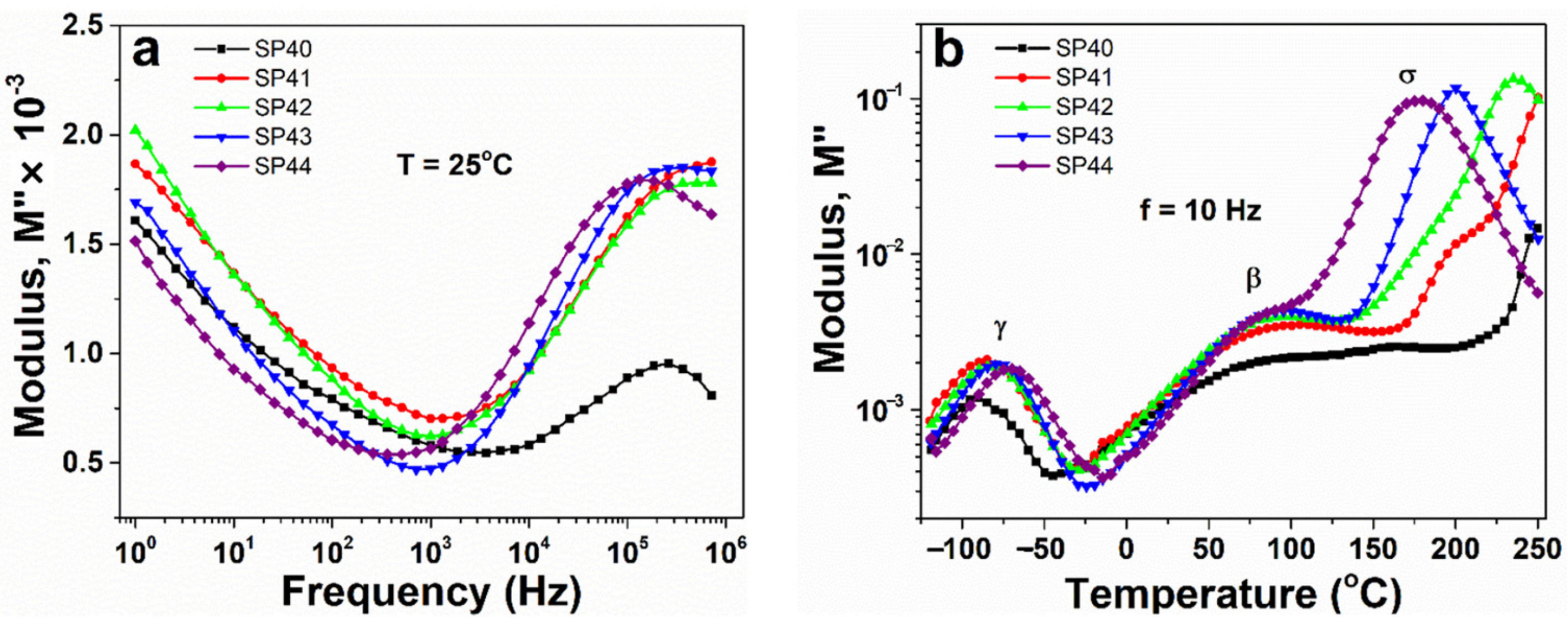
The behavior of modulus (M″) with the applied field frequency at room temperature (**a**) and with temperature at 10 Hz (**b**) for the polyimide films.

The behavior of the electrical modulus, M″, with frequency and temperature is presented in Figure 4a and Figure 4b, respectively. At room temperature, the frequency dependences of the electrical modulus, M″, show the same behavior as in the case of dielectric loss (Figure 4a). At low temperatures, M″ exhibits two distinct regions corresponding to secondary γ- and β-relaxations. With further increases in temperature, conductivity relaxation (σ) is detected for all investigated samples (Figure 4b). The dominant dielectric peak of σ-relaxation depicted at temperatures higher than that of 125 °C correlates well with the continuous increase in *ε*″ detected previously in Figure 3. Noticeably, the σ-relaxation peak is promoted to lower temperatures with the introduction of the azochromophore component. Thus, considering the frequency of 10 Hz as a representative example, the maximum σ-relaxation peak is observed at 235 °C for **SP42**, 201 °C for **SP43**, and 178 °C for **SP44** films. A similar tendency in the results was observed by measuring T_g_ by DSC, as was reported early [30].

**Figure 5:**
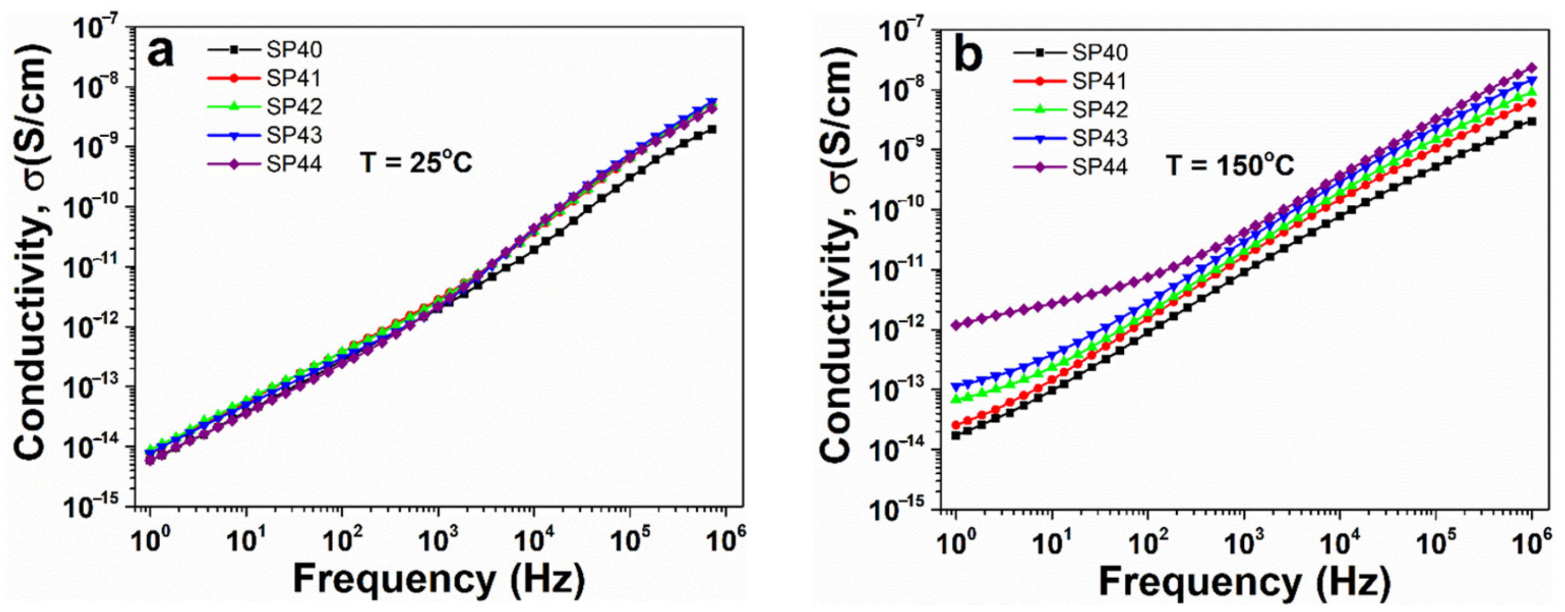
The behavior of conductivity with the applied field frequency at room temperature (**a**) and at 150 °C (**b**) for the polymer films.

Figure 5 presents the evolution of the measured conductivity as a function of frequency at room temperature (a) and at 150 °C (b), respectively. At room temperature, a linear increase in conductivity with increasing frequency was observed for all samples in the entire frequency range. This regime is specific to bound-charge carriers. On the other hand, at a temperature of 150 °C, the conductivity reveals a deviation from a linear increase with frequency. The behavior is specific to the existence of free charges in the material [36]. Following Table 1, the conductivity of supramolecular structures increased by two orders of magnitude, especially for the higher content of azo monomer (**SP44**).

On the other hand, an increase in the contact angle was observed by increasing the content of the azo compound in spite of the increasing polarity of the macromolecular chain, based on the hydrogen linkages that were realized between the OH group from the azo monomer and the imide cycle. As can be seen in Table 2, there is a good correlation between the dielectric constant and the quantity of azo monomer for **SP41**, **SP42**, and **SP43**, which has an increasing tendency, but by introducing much more azochromophore, the dielectric constant decreased but was higher than that of the dielectric constant of the pristine polyimide film, **SP40**.

**Figure 6:**
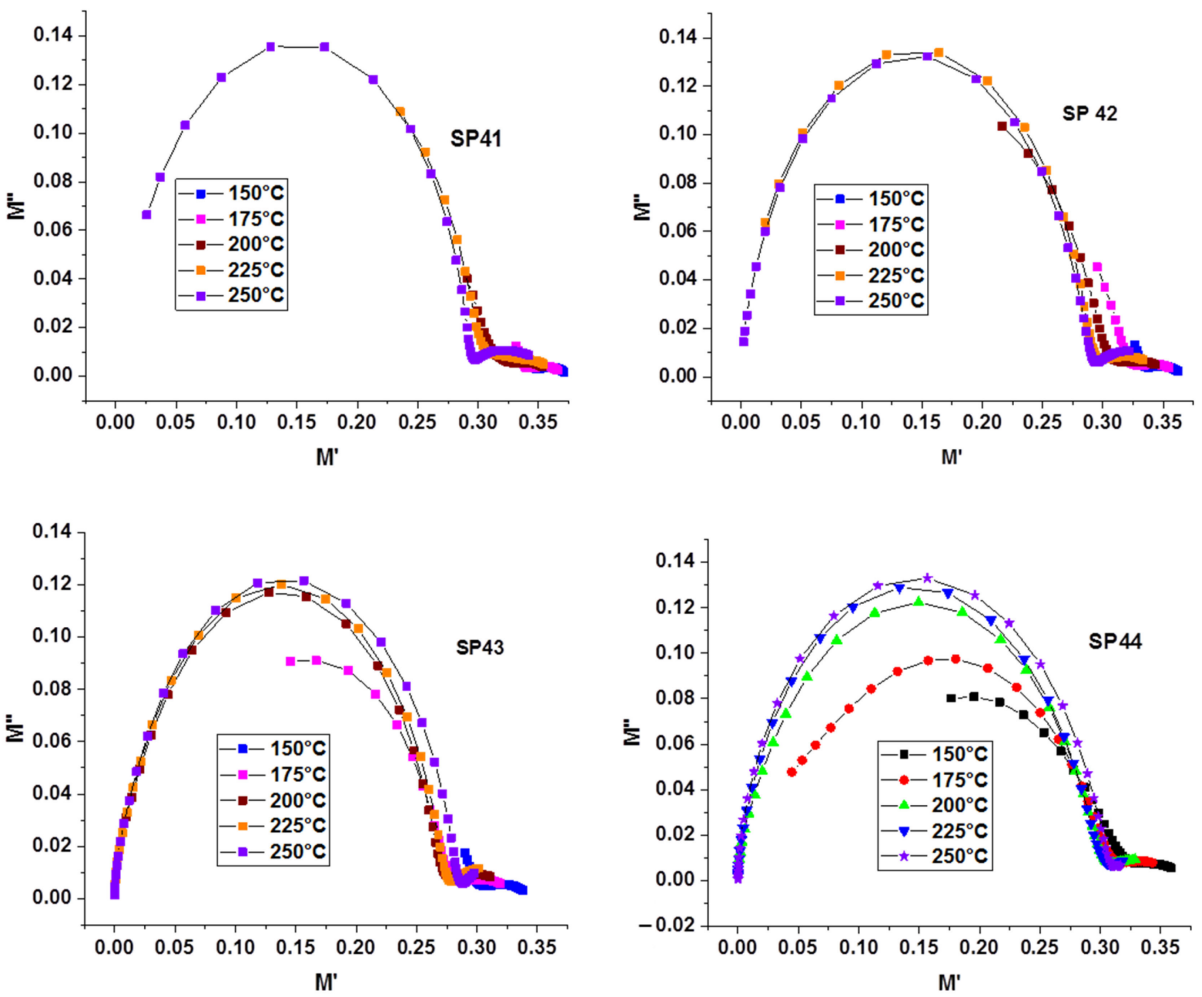
The evolution of Argand curve of **SP41**–**SP44** films at different temperatures.

The Argand plots (M′ versus M″) of the investigated samples were used to see the nature of the relaxation process. As can be seen in Figure 6, all the polymer samples showed semicircular curves, indicating that the relaxation processes follow the Debye model (single relaxation time) [37]. This model presumes that all the dipoles must be identical. For this reason, Debye relaxation is the response of the dielectric relaxation of ideal dipoles to an alternating external electric field. At the same time, by increasing the quantity of azochromophore, the plot maxima increased [38].

**Figure 7:**
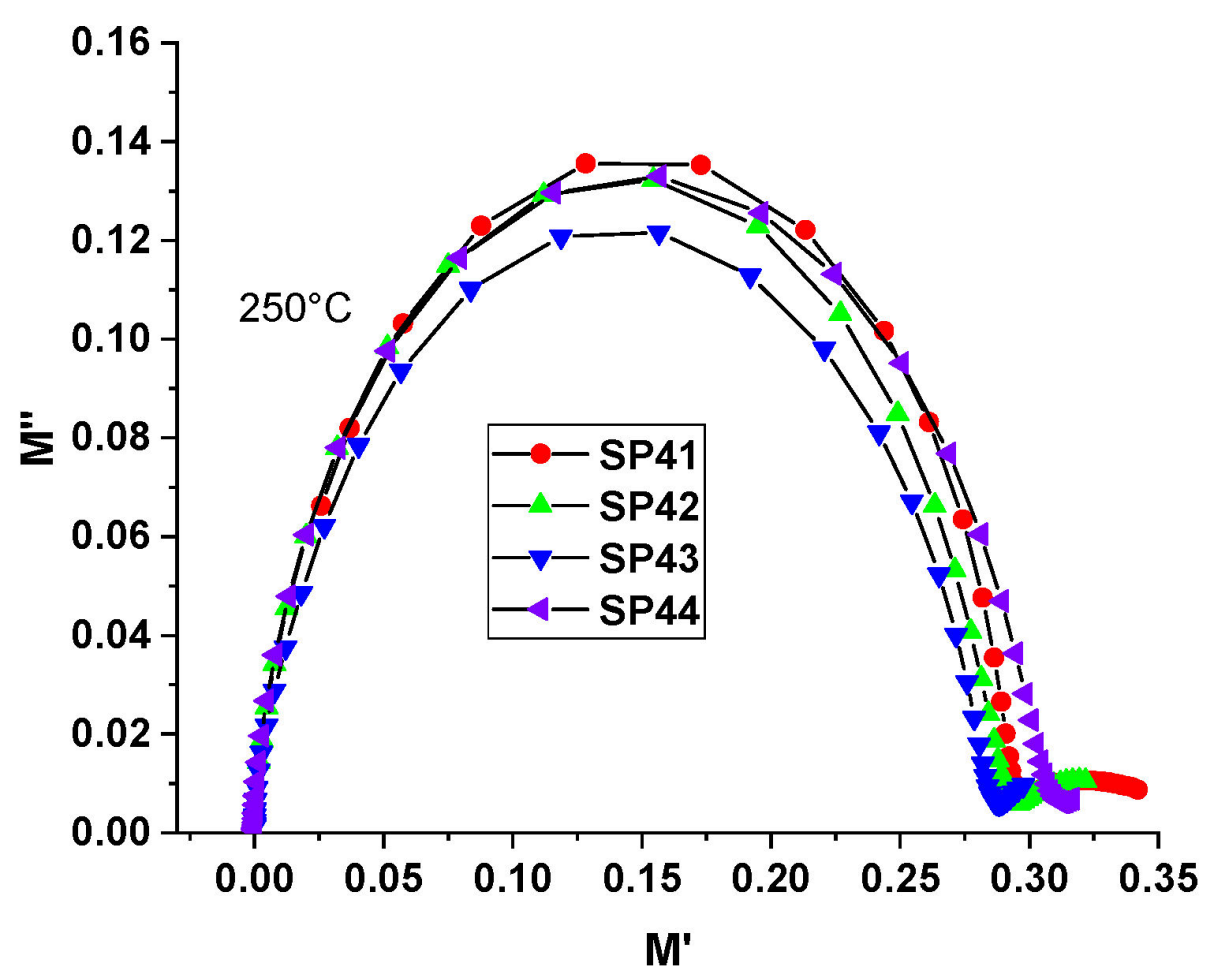
The evolution of Argand curve of **SP41**–**SP44** films at 250 °C.

The Argand plot of the supramolecular structure **SP44** at different temperatures shows that by increasing the temperature, the semicircle becomes more extensive. This means that by increasing temperature over 175 °C, the relaxation processes follow the Debye model (single relaxation time). This behavior was observed for all the supramolecular structures at a very high temperature, 250 °C (Figure 7) [38].

The relationship between the azo monomer quantity and the electrical and mechanical properties can be different due to the connections made between the host (matrix) and the guest (azo monomer). For the dielectric properties, the optimum ratio was 1:0.5. By increasing the quantity of the used azo dye, the value of the dielectric constant slightly decreases. This behavior can be explained by the fact that not all monomer molecules can form hydrogen bonds [6] at high ratios. It seems that some of them remain unlinked in the system. This can influence bulk properties.

### 3.2. Mechanical Properties

The free-standing films demonstrated flexibility, endurance, and integrity despite repeated bending. Their tensile properties have been determined as the averages of three drawing experiments. These supramolecular polyimide films showed similar types of behavior with respect to the elastic deformation range at small strains. By adding the azochromophore, AzoCH_3_, to the polyimide matrix, the mechanical parameters of the supramolecular structures are slightly lower. Thus, the supramolecular structures present values of tensile strength at the break between 95.94 and 121.49 MPa compared to pristine polyimide film, which has a value of 125.31 MPa. The same tendency was observed for the behavior of the elasticity modulus, which has slightly lower values (1.37–1.46 GPa) compared to pristine polyimide film (1.55 GPa). By using a very low quantity of AzoCH_3_, in the case of **SP41**, the value of elongation to break is almost the same as that of pristine polyimide film, around 12.5%. By increasing the quantity of AzoCH_3_, the values of the elongation to break slightly decreased in the range of 9.82–10.65%. The obtained tensile data demonstrated that films with satisfactory mechanical properties (toughness and modulus) can be obtained from all supramolecular structures of polymers that can be further used in various advanced applications.

**Figure 8:**
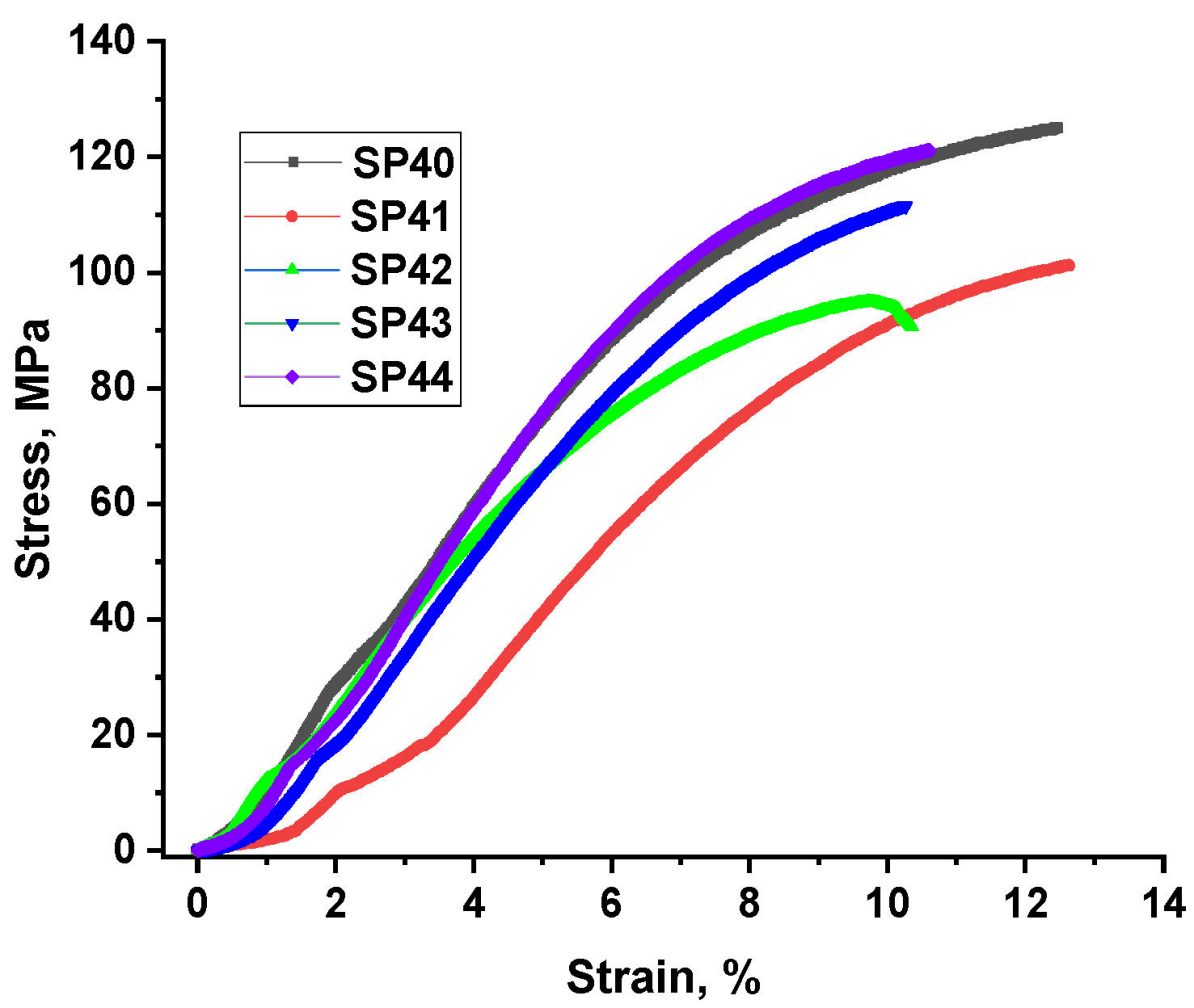
The mechanical properties of the supramolecular structures.

Based on the stress–strain plots from Figure 8, the data from Table 2 were obtained. The results reveal a good correlation between the dielectric constant and the quantity of azo monomer for **SP41**, **SP42**, and **SP43**, which has an increasing tendency, but with the introduction of much more azochromophore, the dielectric constant decreased but is still higher than that of the dielectric constant of the pristine polyimide film, **SP40**.

### 3.3. Surface Wettability and Metal Adhesion

The application of these polymer layers as supports for electrode deposition, as demanded in sensor devices, involved good knowledge of their surface tension properties. These aspects were determined in this work by assessing the interfacial adhesion characteristics of polyimides to platinum. For this scope, the Young–Dupre Equation (1) was utilized:

(1) $$W=2\sqrt{\gamma^{p}\gamma_{Pt}^{p}}+2\sqrt{\gamma^{p}\gamma_{Pt}^{p}}$$

where W is the work of adhesion, $\gamma^{d}$ and $\gamma^{p}$ are the sample disperse and polar surface tension, while $\gamma_{Pt}^{d}$ and $\gamma_{Pt}^{p}$ are the platinum disperse and polar surface tension, respectively.

In order to estimate the work of adhesion at the polymer/Pt interface, it was necessary to evaluate the sample surface energy parameters. This was performed by measuring the contact angles on all films, and the data are introduced in Table 3. Then, the contact angle results were introduced in the Fowkes relation (2) [39] to extract information on the sample’s wettability:

(2) $$1+cos\theta =\frac{2}{\gamma_{l}}\sqrt{\gamma^{p}\gamma_{l}^{p}}+\frac{2}{\gamma_{l}}\sqrt{\gamma^{d}\gamma_{l}^{d}}$$

where θ is the contact angle, the index “l” means the surface tension of the liquid test.

After solving the mathematical system of two equations written for the water and formamide liquid tests, the polar and dispersive properties were obtained for each sample, and the results are listed in Table 3. It was noted that with the incorporation of AzoCH_3_ in the system, the dispersed component of the surface tension decreases for the **SP40**–**SP43** samples, while their surface polar character slightly increases. An exception was observed for the value of γ^d^ of **SP44** film, which increases upon insertion of AzoCH_3_. The introduction of the γ^d^ and γ^p^ in the Young–Dupre Equation (1) allowed determining the work of adhesion with platinum, for which dispersive and polar surface tension values were found to be 22.14 mN/m and 25.29 mN/m, respectively. The magnitude of the W parameter decreased for the **SP41**–**SP44** samples compared to the structure without the azo component, **SP40**. This was probably caused by the tiny increase in the surface polar properties and the concomitant decrease in the dispersive part of γ. In this context, the effects of plasma exposure were also explored to see how the adhesion properties were evolving for each sample. The surface-modified films displayed a reduction of the dispersion feature, whereas the polar components were significantly augmented by about 1.79 times for **SP44** in regard to **SP40**. This is reflected in an enhancement of the work of adhesion with Pt in comparison to the pristine films. Moreover, the films with an increasing AzoCH_3_ content appeared to exhibit, after plasma exposure, more polar groups at the surface, which led to an improvement of the interfacial adhesion with the metal electrode. This will have a positive impact when using these polymers as supports for sensor manufacturing.

### 3.4. Surface Morphology and Metal Adhesion at Nanoscale

**Figure 9:**
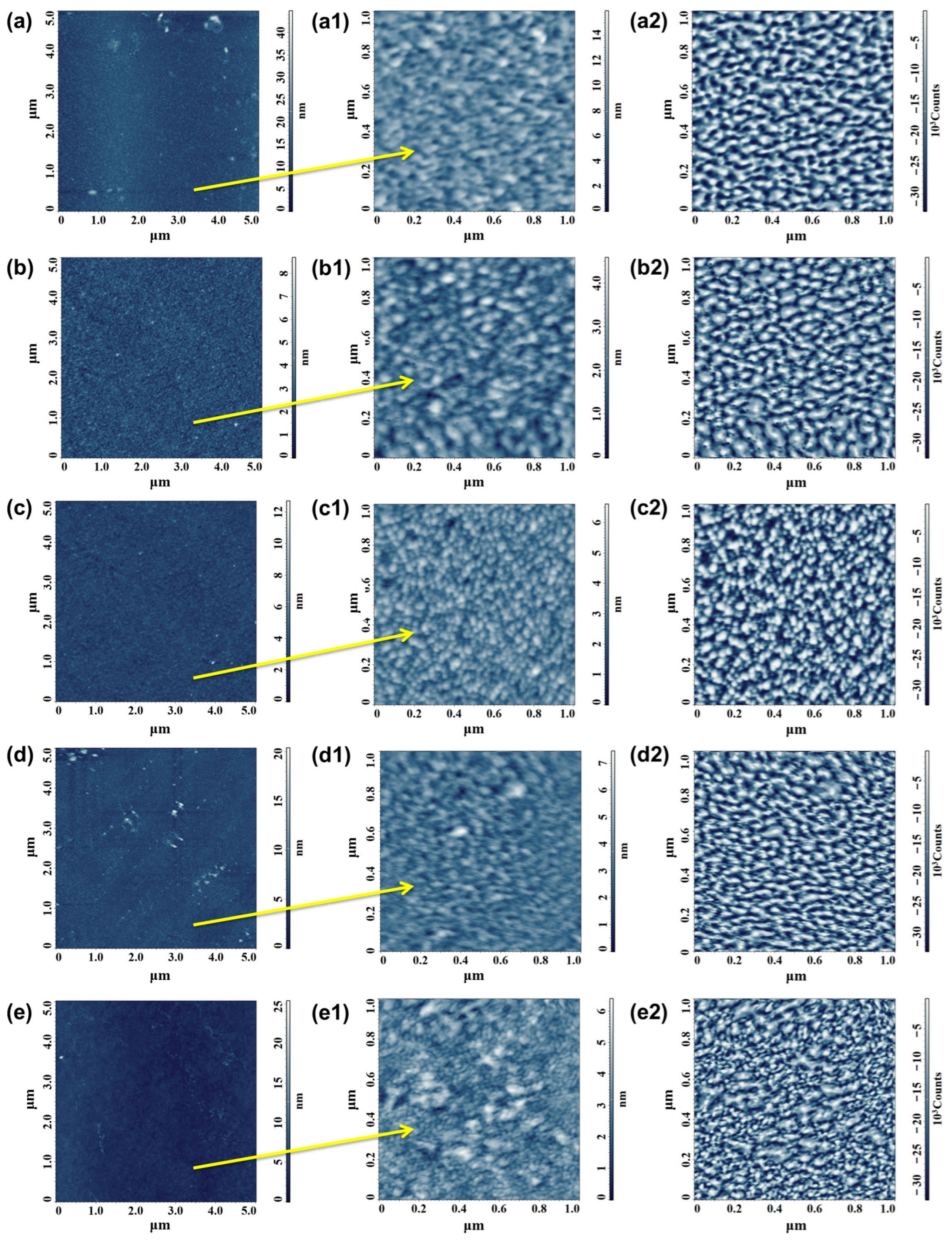
AFM topography images obtained on the surface of the **SP40** (a series), **SP41** (b series), **SP42** (c series), **SP43** (d series), and **SP44** (e series), collected on 5 × 5 μm^2^ (**a**–**e**) and on 1 × 1 μm^2^ (**a1**–**e1**), and the local equalization images obtained by applying the local equalization method on the height images collected on 1 × 1 μm^2^ (**a2**–**e2**).

Atomic force microscopy through the height images obtained on the scanning areas of 5 × 5 μm^2^ (Figure 9a–e) and 1 × 1 μm^2^ (Figure 9(a1–e1)) was used to evaluate the surface morphology dependency on the composition of the polymer, namely the amount of added azochromophore. First, the AFM data collected on 5 × 5 μm^2^ indicates that the peak-to-valley distance (S_y_) for the starting polyimide **SP40** was 44.8 nm, inducing a root mean square roughness (S_q_) of 2.5 nm. The incorporation of even a small amount of azo monomer (1:0.125 molar ratio between polyamidic acid and azo monomer) produced a considerable smoothing of the surface of the **SP41** supramolecular compound film, with S_y_ diminishing to 8.6 nm and leading to a 5-fold decrease in S_q_, to 0.5 nm, compared to the pristine polyimide. It was also found that, with the increase in the amount of azodye in the structure of azo-polyimide supramolecular systems, S_y_ gradually increases from 12.6 nm for **SP42** to 20.3 nm for **SP43**, reaching the case where the molar ratio is 1:1 (**SP44**) even at 25.3 nm. Moreover, the roughness somehow follows a similar path to S_y_, although in this case, the variations are much smaller from one system to another (0.6 nm for **SP42**, 1.1 nm for **SP43**, and 1.2 nm for **SP44**). The lower roughness obtained for the azo-polyimide supramolecular systems can be explained based on the structural organization. The azo group, being in the *trans* configuration, has the tendency to squat. Moreover, the way the dipoles are arranged offers a tightening disposition. All this leads to a flattening and uniformization of the polymer film during the imidization process.

Apparently, on the 5 × 5 μm^2^ scanning area, no special morphological aspects appear, with the surfaces proving to be smooth and uniform. Moving from the microscale analysis to the one at the nanoscale, namely on the 1 × 1 μm^2^ scanning area, the characteristics of the morphological formations are better highlighted, especially in the local equalization images (Figure 9(a2–e2)). The local equalization method involves the processing of an image constituted by a narrow range of intensity values by adjusting the intensities to be better spread on the histogram, employing the full range of intensities equally [40]. This method increases the global contrast, helping the areas of lower local contrast to gain a higher contrast [41,42]. In this way, it is possible to detect different supramolecular organizations at the surface induced by the inclusion of the azo component, depending on its amount.

**Figure 10:**
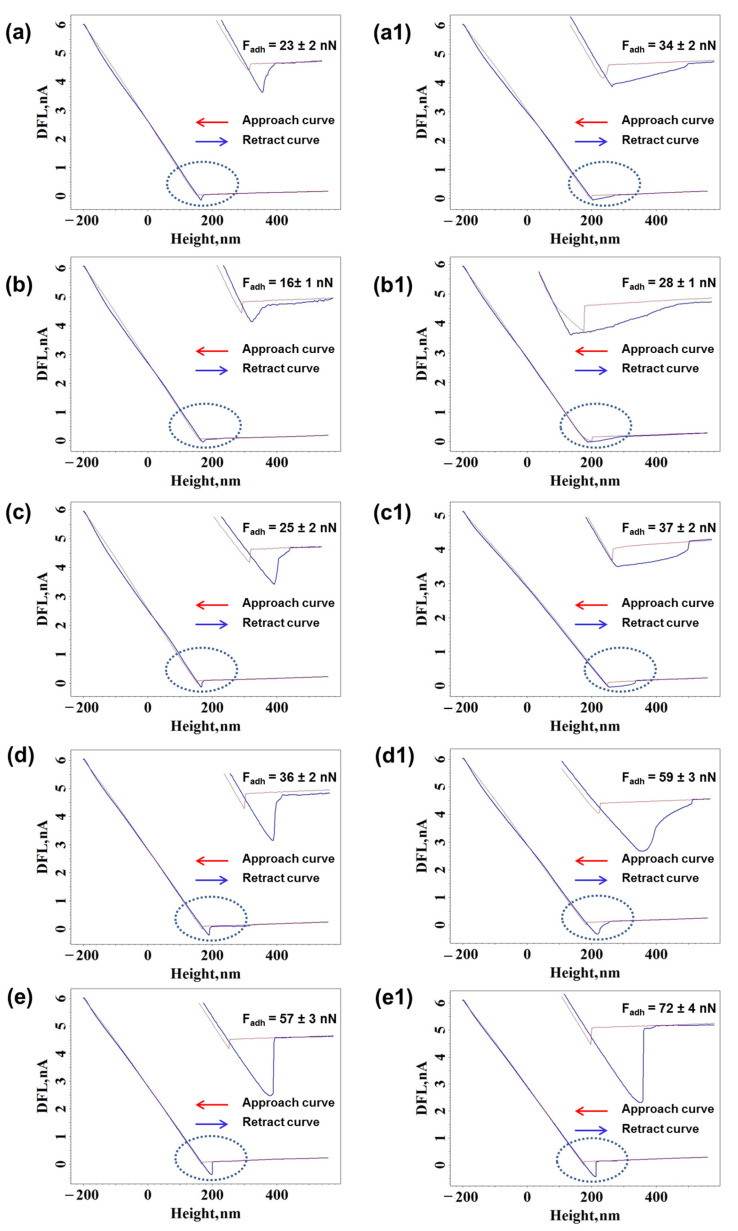
DFL–height curves (approach curve colored in red, retract curve colored in blue) collected for **SP40** (**a**), **SP41** (**b**), **SP42** (**c**), **SP43** (**d**), and **SP44** (**e**), before plasma exposure (**a**–**e**), and after plasma exposure (**a1**–**e1**). Details of the DFL–height curve at the contact point where the adhesion force was measured are presented for each sample.

Since the desired application of these azo-polyimide supramolecular systems is as supports for platinum electrode deposition, as required in sensor devices, the local nanoscale adhesion force between a platinum-covered cantilever’s tip and the studied surfaces was evaluated from the retract DFL–height curves (blue curves from Figure 10) in the contact point. The unmodified control polyimide (**SP40**) showed an adhesion force of 23 nN. When adding a small amount of azo monomer (**SP41**), it initially tends to decrease to around 16 nN, then, with the increase in the molar ratio, an enhancement of the adhesion force at the supramolecular system/platinum interface is observed, even reaching almost 60 nN in the case of the **SP44** sample. This trend can be explained by the intensification of hydrogen bonds with the increase in the molar ratio between polyamide acid and azo dye. The evolution seems to be different compared to the measurements made at the macro level because the physical property is different (work of adhesion in the case of surface wettability measurements and adhesion force in the case of AFM measurements), the determination method is different (calculating the work of adhesion from the evaluation of the sample surface energy parameters and calculating the adhesion force from the DFL–height curve), and the surface on which it is evaluated is different (few mm versus nm). In conclusion, there is an improvement in the ability of platinum to adhere to the studied surface as the amount of the azo component increases. Moreover, it was found that light plasma treatment of the surfaces that are intended to be used as flexible supports for sensors, before the deposition of the electrode, considerably improves the adhesion of the platinum and thus the stability of the deposition [43].

**Figure 11:**
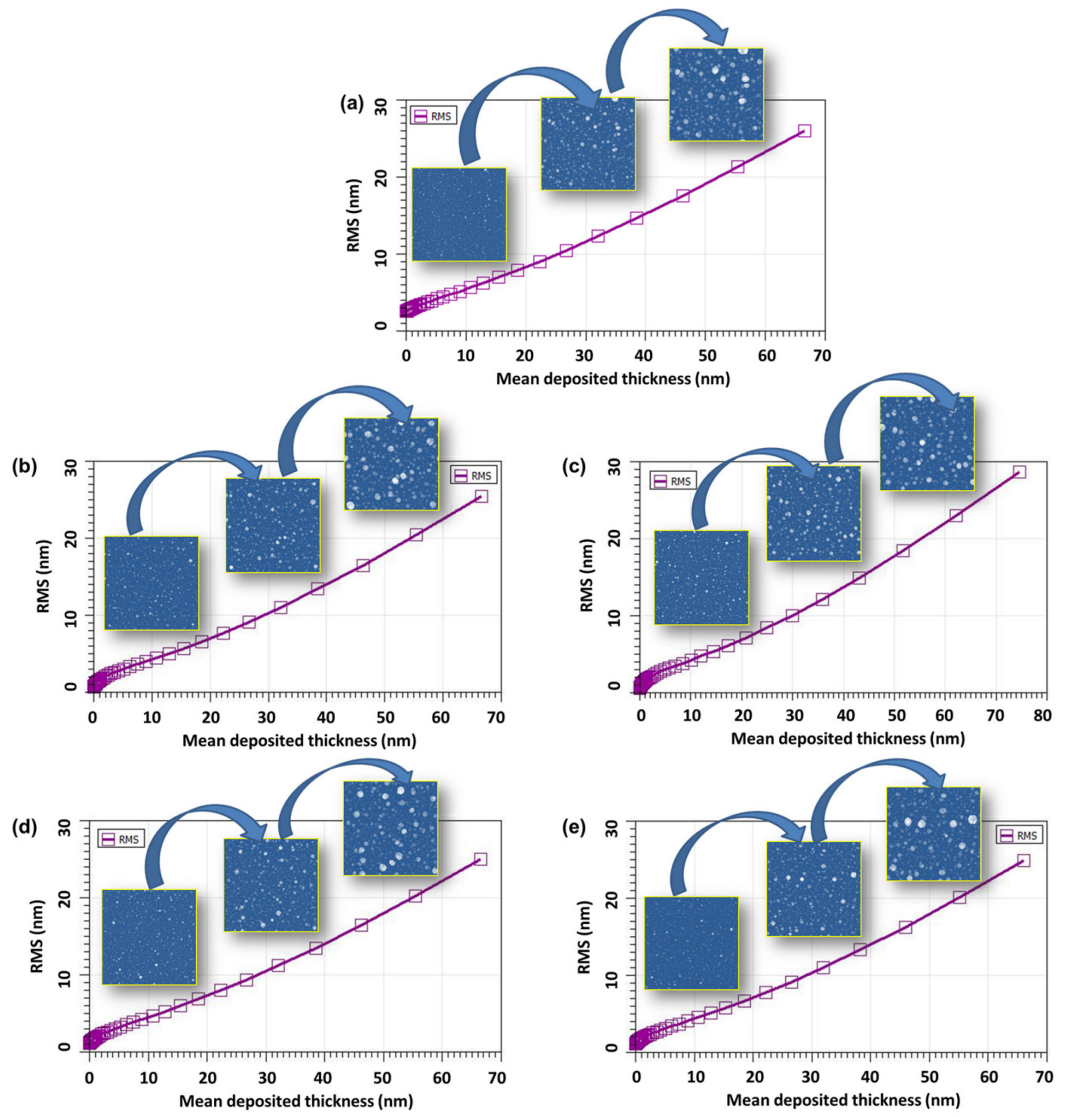
Simulation data of the platinum nanoparticle deposition on the AFM images collected 5 × 5 μm^2^ areas for **SP40** (**a**), **SP41** (**b**), **SP42** (**c**), **SP43** (**d**), and **SP44** (**e**): the evolution of the RMS with respect to the mean deposition thickness, and the aspect of the surface during the deposition stages.

Next, starting from the 2D topographical images obtained on the surface of 5 × 5 μm^2^ for each sample, a simulation of the deposition of platinum nanoparticles was realized. This simulation was made using the Gwyddion 2.56 software using the columnar deposition type. The simulation of columnar film growth employs a Monte Carlo deposition algorithm [44,45]. Thus, small particles of 3 nm in diameter (this dimension was chosen to simulate the dimension of the platinum nanoparticles used in general in electrode deposition) were directed incidentally to the surface by using certain parameters. The particles hit the surface randomly and stick to it, and due to the shading effect, fewer particles will stick to the lower parts of the surface and more particles will grow on the higher parts of the surface, leading to the development of columns, the distribution being isotropic in the horizontal plane [46]. The evolution of the root mean square value of the heights (RMS) with the mean deposited thickness and the aspect of the surfaces during the platinum particle deposition stages is presented in Figure 11. It can be observed that the nanoparticles were uniformly distributed all over the surface, their dispensation depending on the initial surface morphology of the polyimide or azo-polyimide supramolecular systems. It seems that the optimal thickness of the deposition, in which we find a very good coverage of the substrate surface and a monotonous spreading of platinum nanoparticles without the formation of agglomerations, is 30 nm. After this value, the RMS increases suddenly due to the increased crowding tendency. This simulation experiment of the platinum layer growth proves to be very useful in identifying the settings that should be made before the effective deposition of the platinum layer as an electrode for optimal results.

## 4. Conclusions

Materials designed by the host–guest approach, which involved a polyimide precursor and an azochromophore (followed by thermal treatment), resulting in supramolecular systems, were obtained and tested as substrates for sensors based on platinum electrodes.

Dielectric spectroscopy was used to study the characteristics of the supramolecular structures of azo-polyimide films at different temperatures and frequencies. By increasing the quantity of azochromophore, the dielectric constant increased when the ratio between polyamidic acid and chromophore increased from 1:0.125 to 1:0.5. By using a 1:1 ratio, the dielectric constant decreased compared with the one mentioned above but remained at a higher value compared to pristine polyimide film.

The incorporation of the azo monomer with a molar ratio of 1:0.5 increased the dielectric constant of the polyimide by 14% (at room temperature) and the conductivity of charge carriers by two orders of magnitude (at 150 °C, where the conductivity signal of free charge carriers is observed).

The free-standing films were flexible, tough, and remained intact after repeated bending. The obtained tensile data demonstrated that the films with satisfactory mechanical properties realized from all supramolecular structures can be further used in various advanced applications.

The introduction of an azo component was found to reduce the dispersive character while enhancing in a less significant manner the surface polarity of the analyzed films, so the adhesion with Pt was not enhanced. This was solved by plasma exposure of the polymers, which augmented the surface polarity, especially upon AzoCH_3_ addition, and this produced a relevant improvement in the interfacial adhesion. Such results recommend the use of these samples as support for sensor manufacturing.

The AFM measurements reveal that the surface morphology was influenced by the amount of azo compound. The roughness and adhesion force registered at the nanoscale increase as the quantity of the azo dye increases, proving that platinum will adhere better to a surface when the molar ratio between polyamidic acid and azo monomer is 1:1, especially after a short prior plasma treatment. The simulation of the platinum layer growth provided us with information about the deposition parameters that should be used for optimal results.

## Figures and Tables

**Table 1:** Numerical values of dielectric constant, dielectric loss, and conductivity selected at 10 Hz and various temperatures for all investigated samples.

**Polymer**	**ε′**		**ε″**	
	**25 °C**	**150 °C**	**25 °C**	**150 °C**
**SP40**	2.5 ± 0.02	2.7 ± 0.02	6 × 10^−3^ ± 0.1 × 10^−4^	2 × 10^−2^ ± 0.1 × 10^−3^
**SP41**	2.7 ± 0.02	2.9 ± 0.02	10 × 10^−3^ ± 0.1 × 10^−4^	3 × 10^−2^ ± 0.1 × 10^−3^
**SP42**	2.8 ± 0.02	3.0 ± 0.02	10 × 10^−3^ ± 0.1 × 10^−4^	4 × 10^−2^ ± 0.1 × 10^−3^
**SP43**	2.85 ± 0.03	3.3 ± 0.03	9 × 10^−3^ ± 0.1 × 10^−4^	7 × 10^−2^ ± 0.1 × 10^−3^
**SP44**	2.6 ± 0.02	3.4 ± 0.03	6 × 10^−3^ ± 0.1 × 10^−4^	50 × 10^−2^ ± 4 × 10^−3^
**Polymer**	**σ (S/cm)**			
	**25 °C**		**150 °C**	
SP40	3.5 × 10^−14^ ± 0.3 × 10^−15^		9.6 × 10^−14^ ± 0.8 × 10^−15^	
SP41	5.3 × 10^−14^ ± 0.5 × 10^−15^		1.5 × 10^−13^ ± 0.1 × 10^−14^	
SP42	5.8 × 10^−14^ ± 0.5 × 10^−15^		2.3 × 10^−13^ ± 0.2 × 10^−14^	
SP43	5.0 × 10^−14^ ± 0.4 × 10^−15^		3.8 × 10^−13^ ± 0.2 × 10^−14^	
SP44	3.6 × 10^−14^ ± 0.3 × 10^−15^		2.7 × 10^−12^ ± 0.2 × 10^−13^	

**Table 2:** Mechanical properties of the polymer films.

Polymer	Stress (MPa)	Strain (%)	Young Modulus (GPa)
**SP40**	125.31 ± 2.16	12.47 ± 0.43	1.55 ± 0.05
**SP41**	101.46 ± 3.36	12.67 ± 0.32	1.37 ± 0.02
**SP42**	95.94 ± 1.23	9.82 ± 0.31	1.37 ± 0.032
**SP43**	111.64 ± 3.39	10.30 ± 0.46	1.46 ± 0.03
**SP44**	121.49 ± 2.72	10.65 ± 0.26	1.43 ± 0.06

**Table 3:** The average values of water (θ_W_) and formamide (θ_F_) contact angle, disperse (γ^d^) and polar (γ^p^) surface tension, and work of adhesion (W) for untreated and plasma-treated **SP40**–**SP44** films.

	**θ_W_ (°)**				**θ_F_ (°)**	
**Sample**	**U**		**P**		**U**	**P**
**SP40**	58.2 ± 0.45		48.6 ± 0.54		39.6 ± 0.55	36.8 ± 0.45
**SP41**	65.2 ± 0.45		46.0 ± 0.71		54.0 ± 0.71	40.4 ± 0.55
**SP42**	70.8 ± 0.45		39.6 ± 0.54		65.8 ± 0.45	42.0 ± 0.45
**SP43**	73.4 ± 0.55		33.6 ± 0.55		69.0 ± 0.71	38.2 ± 0.45
**SP44**	63.0 ± 0.71		29.6 ± 0.55		51.6 ± 0.55	35.2 ± 0.84
	**γ^d^ (mN/m)**		**γ^p^ (mN/m)**		**W (mN/m)**	
**Sample**	**U**	**P**	**U**	**P**	**U**	**P**
**SP40**	26.43 ± 0.53	20.48 ± 0.64	19.66 ± 0.51	30.47 ± 0.89	92.96 ± 0.31	98.10 ± 0.22
**SP41**	18.36 ± 1.08	15.90 ± 0.27	19.74 ± 0.23	35.92 ± 1.03	85.09 ± 0.22	98.71 ± 0.38
**SP42**	11.27 ± 0.09	10.87 ± 0.50	20.50 ± 0.56	47.35 ± 1.08	77.13 ± 0.76	100.21 ± 0.15
**SP43**	10.23 ± 0.72	10.62 ± 0.42	21.99 ± 0.94	52.33 ± 0.97	75.06 ± 0.41	103.43 ± 0.17
**SP44**	18.89 ± 0.71	10.91 ± 0.48	22.37 ± 0.76	54.51 ± 0.82	86.95 ± 0.49	105.32 ± 0.29

U—untreated sample; P—plasma-exposed sample.

## Data Availability

All data that support the findings of this study are incorporated in the main manuscript of this article. No additional data are available for sharing.
